# Sex-Based Dietary Divergence in Plateau Pikas (*Ochotona curzoniae*) but Not Plateau Zokors (*Eospalax baileyi*)

**DOI:** 10.3390/ani15213216

**Published:** 2025-11-05

**Authors:** Feiyang Xue, Xidong Zhu, Le Qin, Yanjun Guo, Jian Sun, Zhengqian Dang, Limin Hua, Bin Chu, Rui Hua

**Affiliations:** 1 Engineering and Technology Research Center for Alpine Rodent Pest Control of National Forestry and Grassland Administration, Key Laboratory of Grassland Ecosystem of the Ministry of Education, College of Grassland Science, Gansu Agricultural University, Lanzhou 730070, China; 18738373198@163.com (F.X.); zhuxidong0510@163.com (X.Z.); qinle0220@163.com (L.Q.); yanjung2024@163.com (Y.G.); sj_gsau@163.com (J.S.); 16647580974@163.com (Z.D.); hualm@gsau.edu.cn (L.H.); 2 Institute of Grassland Research, Chinese Academy of Agricultural Sciences, Hohhot 010011, China

**Keywords:** plateau pika, plateau zokor, diet composition, sexual segregation, trophic niche

## Abstract

**Simple Summary:**

This study employed DNA metabarcoding combined with a self-constructed plant DNA barcode database to analyze sex-specific dietary composition and trophic niche characteristics in two small mammals, plateau pikas (*Ochotona curzoniae*) and plateau zokors (*Eospalax baileyi*), with contrasting lifestyles and social structures in alpine meadows. For plateau pikas, dietary richness was broadly comparable between sexes, with *Taraxacum mongolicum* the most frequent item (20.23% in females; 28.39% in males). Females nonetheless exhibited significantly greater dietary diversity and niche breadth than males. For plateau zokors, richness was likewise comparable, and *T. mongolicum* again constituted the largest dietary proportion (32.81% in females; 25.27% in males). No significant difference in niche breadth was observed between sexes, and their dietary structures showed a high degree of overlap. These findings suggest that small mammals with different lifestyles and social structures may exhibit divergent patterns of dietary variation between sexes.

**Abstract:**

Quantifying sex-specific dietary differences in small mammals reveals the internal resource allocation mechanisms within a species and provides new insights for ecosystem management and conservation practices. The plateau pika (*Ochotona curzoniae*) and plateau zokor (*Eospalax baileyi*) are dominant small mammals that exhibit distinct lifestyles and social structures on the Qinghai-Tibetan Plateau. Despite the fact that the diets of both species have been extensively studied, sex-specific dietary differences have rarely been investigated. This study employed DNA metabarcoding combined with a self-constructed plant DNA barcode database to analyze the diet composition and trophic niche of male and female plateau pika and plateau zokor during the growing season. The results showed that male and female plateau pika consumed 39 and 37 plant species, respectively, and male and female plateau zokor consumed 38 and 39 plant species, respectively. With respect to the plateau pika, males showed a significantly higher intake of *Phlomoides umbrosa* than females (*p* < 0.05), whereas females consumed a significantly greater proportion of tuberous plants (*p* < 0.05). Females also exhibited a significantly greater dietary diversity and trophic niche breadth than males. But there was no significant difference in dietary diversity and trophic niche breadth between the sexes in the plateau zokor. In conclusion, our results show that dietary differences between males and females depend on each species’ lifestyle. Social, surface-living pikas show apparent sex-based differences, while solitary, underground-living zokors do not.

## 1. Introduction

Small mammals play a crucial role in terrestrial ecosystems worldwide. They are not only an important food source for many predators but also significantly influence plant community structure, seed dispersal, nutrient cycling, and soil physicochemical properties through their foraging behavior [1]. In this group, dietary studies have always been central to understanding niche differentiation, community dynamics, and ecosystem functions. An increasing number of studies indicate that animals often show sex-specific trophic niches, as males and females maximize fitness under different constraints imposed by sexual selection, parental investment, and life-history trade-offs [2,3,4]. Classic theory predicts that the sex under stronger intrasexual competition for mates should accept higher foraging variance or risk to secure the energetic and condition-dependent traits that enhance mating success. In contrast, the sex with greater obligatory parental investment prioritizes consistent intake and safety [5]. These sex-specific optima are further modulated by mating system and social structure (e.g., colonial vs. solitary living) that shape competition, vigilance, and time budgets, thereby altering diet breadth and resource overlap [6]. Consequently, we expect stronger dietary segregation when sociality and surface activity amplify sex differences in risk–reward trade-offs, but weaker segregation when both sexes exploit similar, shielded resources in constrained environments.

In the typical alpine rangeland ecosystem of the Qinghai–Tibetan Plateau (QTP), small mammals are the primary herbivores and play a significant role in rangeland ecological processes [7,8]. Specifically, the plateau pika (*Ochotona curzoniae*) and plateau zokor (*Eospalax baileyi*) are dominant small mammals in this ecosystem and represent two distinct lifestyles and social patterns [8]. The plateau pika is sexually monomorphic in external morphology and body mass, lives diurnally above ground in family groups that occupy exclusive burrow systems [9]. Although direct evidence on its mating system is limited, the absence of sexual dimorphism together with observed male–male aggression and a female-biased adult sex ratio suggests a polygynous social structure. In contrast, the plateau zokor (*Eospalax baileyi*) is a subterranean, predominantly solitary rodent that shows little external sexual dimorphism; it lacks a colonial social organization, and mating occurs underground without persistent multi-female groups [10]. Both sexes experience similar below-ground constraints and mainly exploit roots, rhizomes, and storage organs, including selective harvesting for winter caches, which may limit the scope for sex-biased diets compared with surface-active, colonial species [10]. Reports of explicit dominance hierarchies are scarce, consistent with solitary space use outside the breeding season. When the population size of these two mammals increases significantly, they compete with livestock for forage resources, and behaviors such as soil excavation and mound building further exacerbate alpine meadows degradation; thus, they are regarded as “pests” [11]. Hence, studying their diets is essential to elucidate their roles in alpine rangeland ecosystems. Most existing research has focused on the overall diet of a species or population in the context of seasonal variation. Kang [12] found that the diet of plateau pika varied significantly across different periods, Zhang [13] reported that plateau pika and plateau zokor consumed largely the same plant species but in different proportions, and Zhou [14] observed that the two species exhibited trophic niche differentiation at short temporal scales. However, few studies have addressed sexual segregation in food resource use between plateau pikas and plateau zokors. As primary consumers within the Qinghai–Tibet Plateau food chain, both species exhibit high behavioral plasticity, and external environmental factors can readily induce changes in their foraging strategies [15], indirectly affecting the structure of alpine grassland plant communities. In addition, earlier studies on the diet of small mammals rely on traditional methods such as field observations, the microtissue method of stomach contents, laboratory cage feeding, fecal microtissue analysis, and stable isotope analysis [16]. However, traditional dietary methods have distinct limitations in terms of bias, labor intensity, and taxonomic resolution. DNA metabarcoding offers a more sensitive, efficient, and comprehensive approach to studying mammalian diets. At present, DNA metabarcoding was employed to investigate the trophic niche characteristics of dominant rodents in desert steppe ecosystems [17]. Few studies have yet employed DNA metabarcoding to examine the diet composition of plateau pika and plateau zokor.

Consider the distinct lifestyles and social structure of plateau pika and plateau zokor in the alpine meadow ecosystems of the Qinghai–Tibetan Plateau, a key unresolved question is: do males and females of these two dominant small mammals exhibit differences in diet composition and trophic niche utilization, and if so, are these differences consistent across species with contrasting lifestyles. We hypothesize that sex-specific dietary differences occur in plateau pikas but not in plateau zokors. In surface-dwelling and social plateau pikas, the combination of heterogeneous aboveground resources, sex-related behavioral strategies, and complex social structures enhances dietary divergence between males and females. In contrast, in solitary subterranean plateau zokors, tunnel geometry, high energetic costs of burrowing, and the relative homogeneity of belowground storage organs constrain resource selection, resulting in convergent diets between sexes.

## 2. Materials and Methods

### 2.1. Study Area

**Figure 1:**
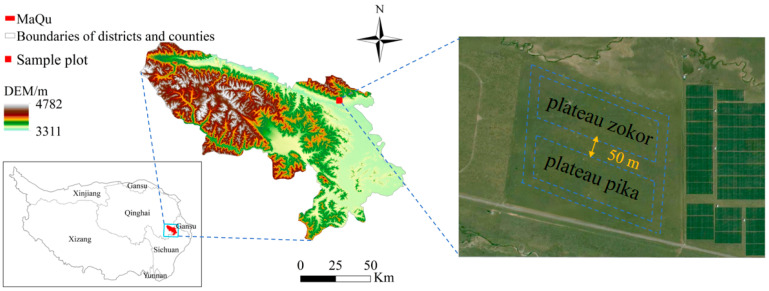
Study area. With the WGS84 datum (coordinate system). Polygons indicate the exact sampling areas. The administrative division base map data used was downloaded from the Standard Map Service System, available at http://bzdt.ch.mnr.gov.cn/, with the approval number: GS (2020) 4619 for the standard map of China. The boundaries of the base map have not been modified. To facilitate comparison with previous work, we note that our site is located ~1 km from the centroid reported by Dong (2025, *Animals* 15:902) [7] and represents a different sampling plot.

The study area is located in Maqu County, Gannan Tibetan Autonomous Prefecture, Gansu Province (Figure 1; 34°0′26″ N, 102°2′47″ E), with an average elevation of 3410 m. The region experiences two distinct seasons: a cold season averaging 314 days and a warm season averaging 51 days. The mean annual temperature is 1.8 °C, with an average annual sunshine duration of 2594.8 h, mean annual precipitation of 615.5 mm, and mean annual evaporation of 1347.3 mm [18]. The rangeland type is alpine meadow. According to our on-site vegetation surveys, the dominant species are herbaceous taxa, namely *Kobresia* spp., *Elymus* spp., *Potentilla* spp., and *Taraxacum* spp. The small mammal community is dominated by plateau pika and plateau zokor. We sampled natural, free-ranging populations within a continuously distributed alpine-meadow landscape. To isolate within-species sex effects and avoid interspecific confounding, we established separate, species-specific 1-ha plots for the two species. To avoid distribution overlap between plateau pika and plateau zokor, adjacent plots were spaced 50 m apart according to the home-range sizes of the two species [19,20]. Field sampling was conducted in August 2024, deliberately scheduled outside the breeding season of both study species—plateau pika and plateau zokor—which generally spans April–June [9,21].

### 2.2. Plant Sampling and Construction of DNA Barcode Database

At each plot, 150 circular quadrats with 0.1 m^2^ were placed at equal 5 m intervals; in total, 300 quadrats were surveyed to record plant species. The results showed that 49 plant species from 40 genera and 19 families were recorded within the plots (Table A1). Guided by the resulting data, plants were collected from the plots, selecting fresh, young leaves with intact petioles. For each species, approximately 1 g of tissue was placed into 5 mL centrifuge tubes, snap-frozen in liquid nitrogen, stored at −80 °C, and transported on dry ice. To ensure high-quality DNA, senescent or severely discolored leaves were avoided. Plant DNA was amplified by PCR using the trnL (UAA) intron primers trnL-c (CGAAATCGGTAGACGCTACG) and trnL-d (GGGGATAGAGGGACTTGAAC), yielding an expected amplicon of approximately 700 bp. DNA extraction and sequencing were outsourced to Personalbio Co., Ltd., Shanghai, China (http://www.personalbio.cn). The resulting plant DNA barcodes were generated and compiled into a reference database.

### 2.3. Classification of Plant Root System Types

Given the contrasting foraging modes of plateau pika (consuming aboveground plant parts) and plateau zokor (consuming belowground parts), the 49 collected plant species were classified into six root system types following Root Systems of Grassland Plants in Northern China [22]: rhizomatous root system type, caespitose root system type, taproot root system type, fibrous root system type, sucker root system type and tuberous root system type.

### 2.4. Animal Sampling and DNA-Based Annotation of Stomach Contents

**Figure 2:**
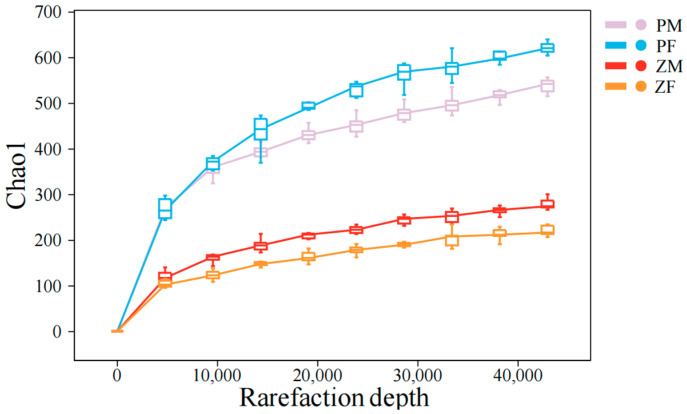
Rarefaction depth. The degree of curve flattening reflects the effect of sequencing depth on the observed sample diversity; the flatter the curve, the more it indicates that the sequencing results sufficiently capture the diversity present in the current samples. ‘PM’ denotes male plateau pika; ‘PF’ denotes female plateau pika; ‘ZM’ denotes male plateau zokor; and ‘ZF’ denotes female plateau zokor.

Within each species-specific 1-ha plot, animals were captured using field randomization guided by current activity signs. Observers walked zig-zag patrols that covered the entire plot and, starting from a coin toss to set the initial bearing, placed one trap at every k-th active sign encountered (pika: active burrow entrance; zokor: fresh mound/runway junction), subject to a ≥10 m minimum spacing between traps and a ≥5 m edge buffer. If a chosen point was unsuitable (bare ground/rock), the station was relocated ≤2 m to the nearest suitable microsite. Traps were checked at dusk and reset at the same station. Using foot-hold restraining traps—padded devices that secure the animal’s foot to limit movement and do not employ break-back/cervical mechanisms—we captured 20 plateau pikas and 20 plateau zokors (40 individuals in total). Subsequently, both plateau pikas and plateau zokors were euthanized under isoflurane inhalation anesthesia [23]. Of these, there were 10 female and 10 male plateau zokors, and 11 female and 9 male plateau pikas. All individuals were dissected, and stomach contents were collected, placed into cryogenic tubes, snap-frozen in liquid nitrogen, and transported on dry ice. The experimental protocol was approved by the Animal Ethics Committee of Gansu Agricultural University (GSAU-Eth-PRA-2023-023) and was carried out in accordance with the relevant ethical guidelines. Stomach-content DNA was amplified by PCR using the trnL (UAA) intron primers trnL-c CGAAATCGGTAGACGCTACG) and trnL-d (GGGGATAGAGGGACTTGAAC) [24]. Primer sequences were trimmed with cutadapt (-O: 10, minimum 10 bp overlap) and reads lacking primer matches were discarded [25]. Paired-end reads were merged with VSEARCH (fastq_mergepairs), quality-filtered (fastq_filter), dereplicated (derep_fulllength), and clustered at 98% sequence identity using cluster_size [26]. Chimeras were detected de novo with UCHIME (uchime_denovo) [27], and any residual putative chimeras were further removed with a custom Perl script to obtain a high-quality read set. The resulting sequences were then clustered at 97% identity with cluster_size to generate representative sequences and an OTU (Operational taxonomic unit) table [26]. Singleton OTUs (i.e., OTUs with a total abundance of 1 across all samples) and their representative sequences were removed. Representative sequences were queried against a curated plant DNA barcode reference database, and taxonomic assignments were made using BitScore as the decision criterion; for each representative sequence, the top-BitScore hit was retained [28]. Based on the OTU abundance table, rarefaction was performed by randomly subsampling each sample to a uniform sequencing depth to estimate the number of observed OTUs and their relative abundances at that depth (pipeline largely following the VSEARCH workflow; analyses supported by Shanghai Personalbio) [29]. DNA sequencing of the stomach contents was also outsourced to Personalbio Co., Ltd., Shanghai, China (http://www.personalbio.cn), and sequence reads were matched against the previously constructed plant DNA barcode database to identify the ingested plant species. Sequencing depth was sufficient to capture the dietary diversity across individuals (Figure 2).

### 2.5. Data Analysis

#### 2.5.1. Food Diversity

We computed dietary indices from the relative abundances of plant food taxa in stomach contents. Species richness was estimated by the Chao1 index (Chao1) [30], which infers the total number of dietary plant taxa, including rare foods detectable at low read counts. Dietary diversity was quantified by the Shannon index (H) [31] and the Simpson index (D) [32], both reflecting how many food taxa are eaten and how consumption is distributed among them (higher values indicate a more varied diet rather than reliance on a few foods). Dietary evenness was measured by Pielou’s index (J) [33], indicating whether intake is evenly spread across foods versus being dominated by a few preferred items (e.g., a diet overwhelmingly composed of one plant shows low evenness).

*S_obs_* = the total number of non-zero OTUs actually observed in the sample (1)

(2) $$\mathrm{Chao}1=S_{\mathrm{obs}}+F_{1}^{2}/{2F}_{2}$$

(3) $$H_{\mathrm{food}}=-\sum_{i=1}^{S}P_{i}\ln P_{i}$$

(4) $$D_{\mathrm{food}}=1-\sum_{i=1}^{S}P_{i}^{2}$$

(5) $$J_{\mathrm{food}}=H_{\mathrm{food}}/\ln(S)$$

(6) $$D_{bc}=\sum_{i=1}^{S}\left|x_{i}-y_{i}\right|/\sum_{i=1}^{S}\left(x_{i}+y_{i}\right)$$

In the formula: *F*_1_ represents the number of OTUs with an abundance of 1; *F*_2_ represents the number of OTUs with an abundance of 2; *S* represents the number of OTUs in the sample; *Pᵢ* represents the relative abundance of the *i*-th OTU in the sample. 

Bray–Curtis distance is a commonly used metric for assessing differences in species composition between two samples, especially in ecology for community comparisons. It primarily calculates the differences by considering species abundance in each sample, making it particularly suitable for analyzing relative abundance and community composition differences [34].

#### 2.5.2. Trophic Niche Breadth and Overlap

To characterize feeding niches, we used the Levins index (*B*) [35] to describe trophic niche breadth—higher values indicate consumption of many food taxa at more similar shares (a broad, generalist diet), whereas lower values indicate focus on few preferred foods (a specialist diet). Pairwise trophic niche overlap between groups was quantified by Pianka’s overlap index (*O_jk_*) [36], which measures the similarity of food-use profiles; values close to 1 denote highly similar diets, and values near 0 denote distinct diets.

(7) $$B=1/\sum P_{j}^{2}$$

In the formula, *B* represents niche breadth, and *P_j_* represents the relative abundance of food item *j*.

(8) $$O_{\mathrm{jk}}=\sum (P_{\mathrm{ij}}\cdot P_{\mathrm{ik}})/\sqrt{\sum \left(P_{\mathrm{ij}}^{2}\right)\cdot \sum \left(P_{\mathrm{ik}}^{2}\right)}$$

In the formula, *P_ij_* and *P_ik_* represent the relative abundance of food item *i* in the diets of species *j* and *k*, respectively. The value of *O_jk_* ranges from 0 to 1. According to Krebs’ criterion, *O_jk_* > 0.3 is considered a meaningful overlap, and *O_jk_* > 0.6 is considered a significant overlap.

### 2.6. Statistical Analysis

First, the raw data were preprocessed in Excel 2021, including data cleaning and formatting, to ensure accuracy and consistency. The relative abundance of OTUs within each sample was used to calculate the relative abundances of plant species in the stomach contents. Using QIIME2 2022.11, rarefaction curves were generated by randomly subsampling the total sequence counts in each sample at different depths, plotting the number of sequences obtained and the corresponding OTU counts at each depth [37]. Data were analyzed in SPSS 25.0. We now specify the tests and software used to check assumptions. Normality was evaluated with the Shapiro–Wilk test and visualized using Q–Q plots against the theoretical normal distribution N (μ, σ^2^), with μ and σ estimated from the sample within each group [38], and homoscedasticity with Levene’s test (median-centered). Independent-sample *t*-tests (under normality) or Mann–Whitney U tests (otherwise) were used to assess differences in plant community characteristics among sites and diet composition between plateau pikas and plateau zokors. Bray–Curtis distances were calculated using the vegan package in R 4.5.1 [34], and non-metric multidimensional scaling (NMDS) was performed based on the Bray–Curtis distance matrix to evaluate differences in stomach content food assemblies of plateau pika and plateau zokor. Data visualization of the analysis results was performed using Origin 2021.

## 3. Results

### 3.1. Diet Composition and Trophic Niche Characteristics of Plateau Pika

#### 3.1.1. Sex-Specific Diet Composition and Foraging Proportions

**Figure 3:**
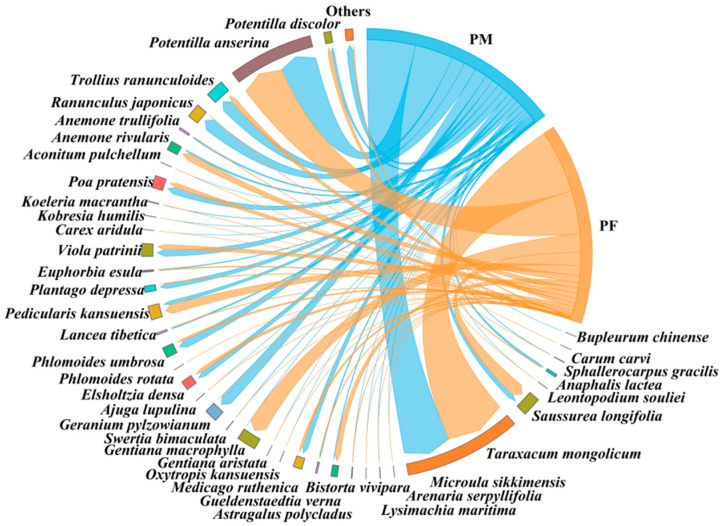
Diet composition of male and female plateau pika. ‘PM’ denotes male plateau pika, ‘PF’ denotes female plateau pika.

The results showed that male and female plateau pika consumed a total of 39 and 37 plant species, respectively (Figure 3). Two taxa were detected exclusively in males *Arenaria serpyllifolia* (0.16 ± 0.10%; all variables are reported as mean ± SE.) and *Lysimachia maritima* (0.06 ± 0.05%). The top five plant species by relative abundance in males were *Taraxacum mongolicum* (28.39 ± 9.33%), *Geranium pylzowianum* (11.66 ± 4.78%), *Poa pratensis* (8.87 ± 3.57%), *Astragalus polycladus* (7.04 ± 2.07%), and *Sphallerocarpus gracilis* (6.14 ± 3.56%). In females, the top five were *Taraxacum mongolicum* (20.23 ± 3.38%), *Potentilla anserina* (11.46 ± 4.33%), *Potentilla discolor* (9.18 ± 4.40%), *Poa pratensis* (8.55 ± 1.92%), and *Astragalus polycladus* (6.53 ± 1.99%). Males showed a significantly higher intake of *Phlomoides umbrosa* than females (Nonparametric Mann–Whitney U test, *p* < 0.05) (Table 1), whereas females consumed a significantly greater proportion of tuberous plants (Nonparametric Mann–Whitney U test, *p* < 0.05) (Table 2). 

**Figure 4:**
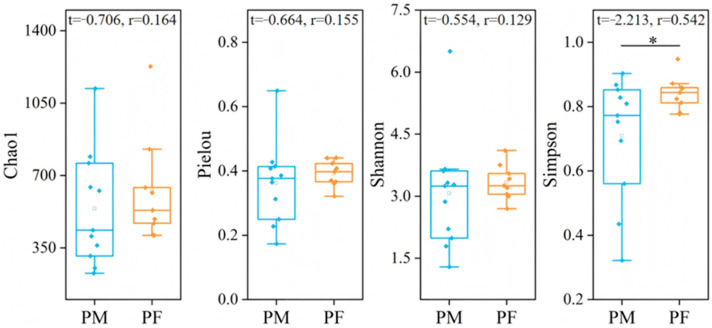
Differences in dietary diversity between male and female plateau pika. ‘PM’ denotes male plateau pika, ‘PF’ denotes female plateau pika. The central line indicates the median; boxes represent the interquartile range (IQR, 25th–75th percentiles); whiskers extend to the most extreme values within 1.5 × IQR; and points denote individual observations. “*” indicates a significant difference at the 0.05 level. The same boxplot definitions apply to all subsequent figures.

Analysis of plateau pika dietary diversity (Figure 4) revealed that the Chao1 index exhibited a wider range of variation in males, whereas females showed relatively concentrated values, indicating greater fluctuations in community species richness among males compared with females. Overall, females exhibited higher Pielou’s evenness indices than males, with lower dispersion, suggesting a more balanced consumption of plant species. The Simpson index was significantly higher in females than in males (independent-samples *t*-test, *p* < 0.05), indicating a higher number and greater evenness of consumed species.

#### 3.1.2. Sex-Specific Trophic Niche

**Figure 5:**
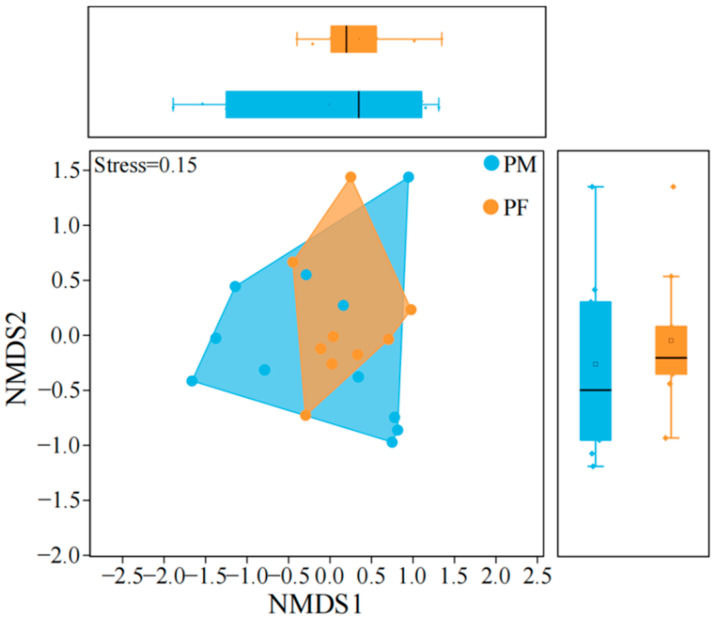
Trophic niche structure model of male and female plateau pika. ‘PM’ denotes male plateau pika, ‘PF’ denotes female plateau pika. Boxplots summarize the distributions of animal sample scores on NMDS1 and NMDS2, and their axis ticks are aligned with those of the main NMDS plot. The same applies hereinafter.

Analysis of the trophic niche characteristics of plateau pika (Table 3, Figure 5) revealed that females exhibited a significantly greater niche breadth than males (Nonparametric Mann–Whitney U test, *p* < 0.05), indicating broader food utilization. Despite this difference, niche overlap between sexes was high, suggesting that the principal plant taxa consumed were largely shared. here were significant differences in niche breadth between sexes, the degree of overlap was high, suggesting that while males and females differ in diet breadth, the main plant species they consume remain largely similar. Non-metric multidimensional scaling (NMDS) of dietary structure showed a certain degree of separation between male and female plateau pika, with partial overlap. Male sample points were more widely dispersed, reflecting high individual variability in food use, whereas female sample points were relatively clustered, indicating a more stable foraging strategy.

### 3.2. Diet Composition and Trophic Niche Characteristics of Plateau Zokor

#### 3.2.1. Sex-Specific Diet Composition and Foraging Proportions

**Figure 6:**
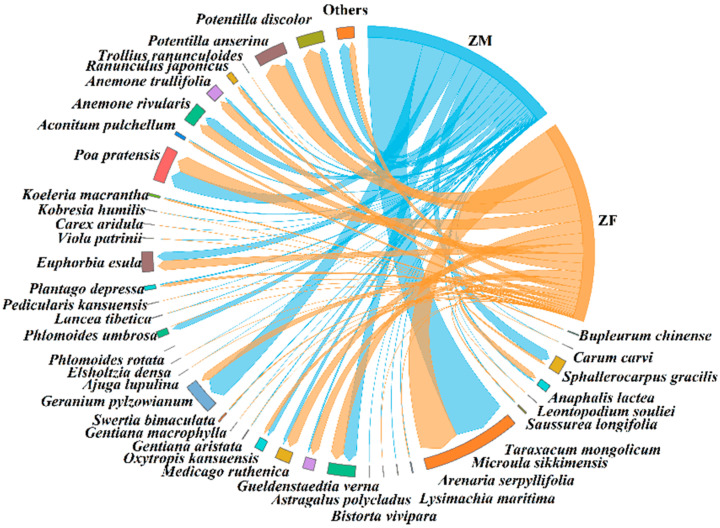
Diet composition of male and female plateau zokor. ‘ZM’ denotes male plateau zokor, and ‘ZF’ denotes female plateau zokor.

The study found that male and female plateau zokors consumed 38 and 39 plant species, respectively (Figure 6). Compared to females, males additionally consumed *Bupleurum chinense* (0.02 ± 0.01%), whereas females additionally consumed *Carum carvi* (0.002 ± 0.001%) and *Phlomoides rotata* (0.0004 ± 0.0001%) relative to males. The top five plant species consumed by males were *Taraxacum mongolicum* (25.27 ± 9.55%), *Potentilla anserina* (19.75±5.46%), *Ranunculus japonicus* (6.99 ± 3.90%), *Trollius ranunculoides* (6.48 ± 6.08%), and *Geranium pylzowianum* (6.45 ± 5.92%). For females, the top five were *Taraxacum mongolicum* (32.81 ± 10.91%), *Potentilla anserina* (22.43 ± 7.73%), *Gentiana macrophylla* (9.65 ± 6.35%), *Saussurea longifolia* (6.91 ± 4.79%), and *Pedicularis kansuensis* (4.95 ± 4.95%). Females consumed significantly more *Gentiana macrophylla* than males (Nonparametric Mann–Whitney U test, *p* < 0.05) (Table 4). No significant differences were observed between sexes in the consumption proportions of plants with different root types (Nonparametric Mann–Whitney U test) (Table 5).

**Figure 7:**
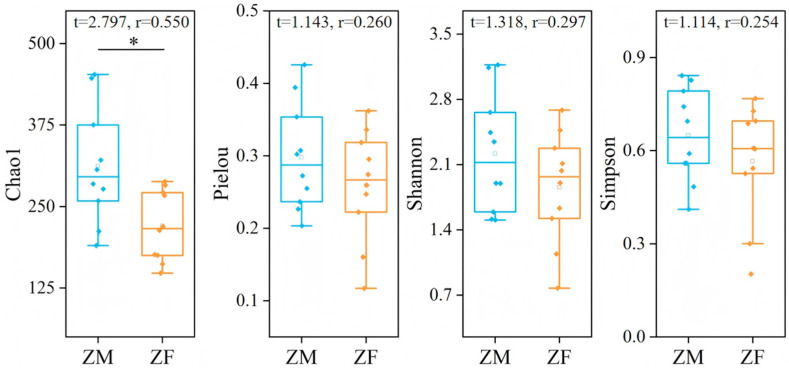
Differences in dietary diversity between male and female plateau zokor. ‘ZM’ denotes male plateau zokor, and ‘ZF’ denotes female plateau zokor. “*” indicates a significant difference at the 0.05 level.

The Chao1 index, Pielou’s evenness index, Shannon–Wiener index, and Simpson index for male plateau zokors were all higher than those for females, but only the Chao1 index showed a statistically significant difference (Independent-sample *t*-tests, *p* < 0.05). The Pielou evenness index and the Shannon and Simpson diversity indices showed no significant differences between male and female plateau zokors (Independent-sample *t*-tests, *p* > 0.05) (Figure 7).

#### 3.2.2. Sex-Specific Trophic Niche

**Figure 8:**
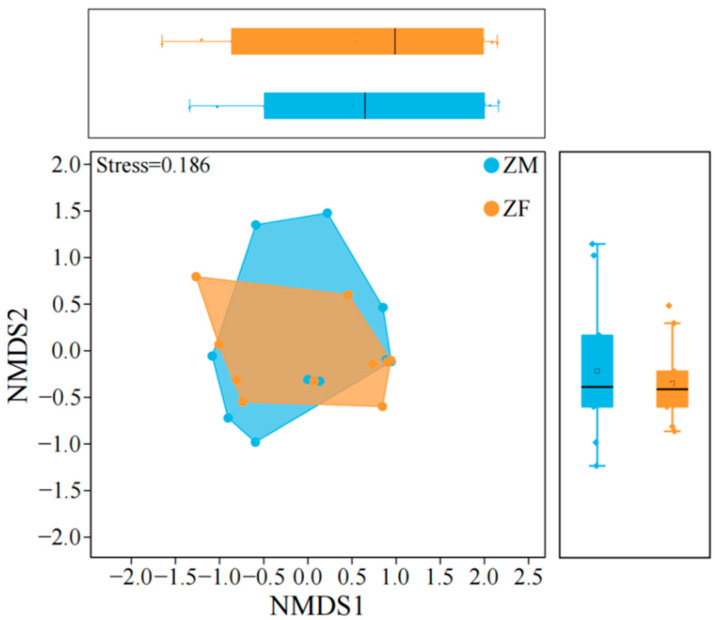
Trophic niche structure model of male and female plateau zokor. ‘ZM’ denotes male plateau zokor, and ‘ZF’ denotes female plateau zokor.

Analysis of the trophic niche characteristics of plateau zokor (Nonparametric Mann–Whitney U test, *p* > 0.05) (Table 6, Figure 8) revealed no significant sex differences in niche breadth, and niche overlap was high, indicating that males and females consumed largely the same plant taxa. NMDS ordination of diet composition likewise revealed substantial intersex overlap. Male samples were more widely dispersed in ordination space, consistent with greater interindividual variability in resource use, and the male niche polygon nearly encompassed that of females.

## 4. Discussion

### 4.1. Sex-Specific Dietary Differences in Plateau Pikas and Plateau Zokors

We found clear sex-specific dietary differences in plateau pikas but not in plateau zokors. Male plateau pikas consumed more *Phlomoides umbrosa*. As a fibrous-rooted plant, it contains a higher proportion of structural carbohydrates, resulting in lower digestibility and energy density [39]. Meanwhile, species of the genus Phlomoides are characterized by high levels of trace elements and pharmacologically active constituents, which may play key roles in the growth and development of plateau pikas [40]. By contrast, female plateau pikas consumed more tuberous-rooted plants, which typically have higher starch contents and energy density, more readily meeting their energetic demands. Given the pika’s sexually monomorphic morphology, colonial sociality, and polygynous mating system, this pattern accords with the general principle that males more often target high-energy yet scarce resources, while females prioritize stable, secure, and nutritionally balanced foods because of higher physiological costs. By contrast, female plateau pikas consumed more tuberous-rooted plants, which typically have higher starch contents and energy density, thereby better meeting their energetic demands [41]. Although direct behavioral evidence is lacking, the species is presumed to exhibit a polygynous mating tendency based on observed male–male aggression and a female-biased adult sex ratio [9]. Given the pika’s sexually monomorphic morphology and colonial social organization, this dietary pattern likely reflects sex-specific energetic requirements rather than a fixed preference for resource type. The present study was conducted during the peak plant-growing season (August), corresponding to the late reproductive period when male energetic expenditure on mating declines, whereas females remain under higher energetic demand due to lactation and post-reproductive recovery. This context may explain their greater consumption of starchy, energy-rich foods [42,43]. In zokors, however, sex-related differences were weak. Their subterranean lifestyle imposes strict energetic and geometric constraints on foraging, and the roots and rhizomes they consume are relatively homogeneous and substitutable [44]. Moreover, their solitary social structure reduces competition within the species. These factors together explain the convergence in diet between male and female zokors [45].

### 4.2. Sex-Specific Food Diversity and Trophic Niche in Plateau Pikas and Plateau Zokors

Food diversity and niche breadth are classic proxies for adaptive foraging under optimal/central-place foraging and sex-based segregation frameworks, which predict that reproductive roles, movement ecology, and resource spatiotemporal variance jointly shape diet composition [46,47,48,49]. In this study, female plateau pikas exhibited significantly higher Simpson diversity and a broader trophic niche breadth than males. This indicates a more even use of multiple food items and a reduced dependence on top-preferred foods. Such a pattern aligns with the view that, during the post-reproductive period—encompassing body-composition recovery—females face elevated demands for energy and micronutrients and therefore diversify their diets to hedge against resource shortfalls (a risk-spreading/even-foraging strategy) [50,51]. By contrast, males showed larger fluctuations in food richness, consistent with broader spatial sampling and episodic targeting of patchy, high-payoff items under higher activity and competition, as predicted by sex segregation/competitive-ability hypotheses [46,52]. For plateau zokors, the pattern was reversed: males displayed higher dietary diversity and richness than females, which we attribute to larger activity areas, larder-hoarding tendencies, and complex burrow architecture in male subterranean mammals, all of which expand the underground encounter set [44,53,54]. Nevertheless, NMDS ordination revealed greater niche overlap and dietary convergence between the sexes in zokors. This phenomenon is parsimoniously explained by their subterranean lifestyle: high excavation costs [55,56,57], negligible aboveground predation risk [58], and the comparatively stable composition and availability of underground foods (storage roots/tubers) together constrain profitable dietary divergence between the sexes [44,59]. Despite these sex-specific differences in dietary diversity and composition, both species exhibited high Pianka overlap indices (0.87 in plateau pikas and 0.91 in plateau zokors), indicating substantial sharing of plant resources between males and females. This apparent coexistence of sex-based dietary divergence and high overall overlap is not contradictory: it reflects differences in foraging strategy rather than complete segregation of food resources [60]. In plateau pikas, the sexes utilize similar food types but in different proportions and combinations, resulting in moderate niche differentiation within a largely common resource pool. In plateau zokors, by contrast, their subterranean lifestyle and the homogeneity of underground foods constrain the opportunity for such divergence [61,62], producing both weak sex-specific variation and very high overlap. These findings highlight that trophic niche segregation and overlap can coexist across scales—divergence at the level of resource use strategy may still occur within a shared ecological base [49].

### 4.3. Revisiting Diet Composition of Plateau Pikas and Zokors with DNA Metabarcoding

Food resources are essential for sustaining animal populations, and any shift in dietary composition at a given trophic level can cascade through the entire food web [63]. Our results revealed a broader dietary spectrum for plateau pikas and plateau zokors compared with previous reports based on traditional methods [12,13,64,65]. Earlier studies generally identified 15–26 food species, with pikas showing strong reliance on Poaceae and zokors on forbs such as *Polygonum viviparum* and *Potentilla anserina*. In contrast, we detected 37–39 plant species per sex in both species, with greater use of Asteraceae and Rosaceae and weaker preference for Poaceae. These discrepancies may be attributed to variations in plant community structure across different sites. The higher dietary diversity observed in this study is largely attributable to DNA metabarcoding, which provides superior taxonomic resolution and efficiency relative to direct observation and microhistological analysis. Traditional approaches are prone to bias and systematic error due to subjective identification and low resolution. By contrast, DNA metabarcoding captures rare or cryptic taxa, allowing for a more comprehensive view of mammal diets. To connect taxonomic breadth to functional nutrient intake, future work (or parallel measurements) can integrate bulk stable isotope analyses (e.g., δ^13^C, δ^15^N) of consumer tissues and candidate plants [66,67]. Isotopes trace assimilated carbon and nitrogen over integrative time windows, enabling tests of whether the increased use of Asteraceae/Rosaceae inferred by DNA translates into a more C_3_-plant–like δ^13^C signature and whether sex-specific diets produce detectable differences in δ^15^N (nutritional routing/trophic position) [66,68]. Combining DNA-based relative read abundance with isotope mixing models (e.g., MixSIAR) thus links “what is eaten” to “what is assimilated,” providing a functional complement to our compositional results [69,70].

## 5. Conclusions

Across two sympatric alpine herbivores, we found a consistent contrast: plateau pikas exhibit clear sex-based dietary divergence, whereas plateau zokors show strong intersex dietary convergence and niche overlap. This contrast aligns with their differing lifestyles and resource regimes—heterogeneous, surface-available foods and social structure in pikas versus comparatively stable underground resources and solitary living in zokors—indicating that lifestyle and resource stability govern the scope of intersexual niche differentiation within the same ecosystem.

## Figures and Tables

**Table 1:** Major food items and proportions with significant differences between male and female plateau pika.

Plant Species	Male Plateau Pika (%)	Female Plateau Pika (%)	*U*	*r*	*p*
*Sphallerocarpus gracilis*	6.14 ± 3.56	1.06 ± 0.53	30	0.331	0.152
*Taraxacum mongolicum*	28.39 ± 9.33	20.23 ± 3.38	47	0.042	0.882
*Astragalus polycladus*	7.04 ± 2.07	6.53 ± 1.99	49	0.008	0.997
*Gueldenstaedtia verna*	0.91 ± 0.37	4.49 ± 2.50	33	0.280	0.230
*Medicago ruthenica*	1.91 ± 1.12	5.35 ± 2.48	29	0.348	0.131
*Geranium pylzowianum*	11.66 ± 4.78	3.86 ± 2.10	27.2	0.382	0.095
*Phlomoides umbrosa*	3.15 ± 2.52	0.05 ± 0.02	18	0.535	0.016
*Euphorbia esula*	4.75 ± 2.03	5.06 ± 3.31	38.1	0.195	0.412
*Poa pratensis*	8.87 ± 3.57	8.55 ± 1.92	37.2	0.212	0.370
*Anemone rivularis*	4.82 ± 3.22	4.94 ± 1.78	30.1	0.331	0.152
*Anemone trullifolia*	1.59 ± 0.48	4.50 ± 1.81	32	0.297	0.201
*Potentilla anserina*	3.66 ± 1.48	11.46 ± 4.33	27.5	0.382	0.095
*Potentilla discolor*	3.94 ± 1.37	9.18 ± 4.40	35.1	0.246	0.095

Note: All data are expressed as mean ± standard error (SE). “%” denotes the proportion of this food in the overall diet. The same applies hereinafter.

**Table 2:** Proportion of consumption of plants with different root types by male and female plateau pika.

Root Type	Male Plateau Pika (%)	Female Plateau Pika (%)	*U*	*r*	*p*
Rhizomatous root system type	30.57 ± 5.44	30.62 ± 4.49	49	0.008	0.998
Caespitose root system type	0.76 ± 0.27	0.64 ± 0.25	48	0.025	0.941
Taproot root system type	45.42 ± 7.59	34.66 ± 4.86	40	0.161	0.503
Fibrous root system type	4.15 ± 3.03	1.88 ± 1.23	43	0.110	0.656
Sucker root system type	4.85 ± 2.01	5.09 ± 3.31	37	0.212	0.370
Tuberous root system type	8.92 ± 2.89	23.98 ± 5.33	16	0.569	0.010
Others	5.32 ± 4.05	3.12 ± 1.54	44	0.093	0.710

**Table 3:** Trophic niche breadth and overlap of male and female plateau pika.

	Male Plateau Pika	Female Plateau Pika	*p*
Trophic niche breadth	4.20 ± 0.60	6.27 ± 0.57	0.038
Trophic niche overlap	0.87		-

Note: “-” indicates no data. The same applies hereinafter.

**Table 4:** Major food items and proportions with significant differences between male and female plateau zokor.

Plant Species	Male Plateau Zokor (%)	Female Plateau Zokor (%)	*U*	*r*	*p*
*Saussurea longifolia*	2.72 ± 2.56	6.91 ± 4.79	43	0.118	0.631
*Taraxacum mongolicum*	25.27 ± 9.55	32.81 ± 10.91	45.1	0.085	0.739
*Astragalus polycladus*	0.32 ± 0.27	2.86 ± 2.53	48.2	0.034	0.912
*Medicago ruthenica*	2.30 ± 1.75	1.33 ± 0.71	38	0.203	0.393
*Gentiana macrophylla*	0.05 ± 0.05	9.65 ± 6.35	21	0.506	0.029
*Geranium pylzowianum*	6.45 ± 5.92	0.05 ± 0.02	30	0.338	0.143
*Elsholtzia densa*	2.77 ± 2.76	1.88 ± 1.26	36	0.244	0.315
*Phlomoides umbrosa*	4.07 ± 2.21	1.09 ± 0.87	48.3	0.034	0.912
*Pedicularis kansuensis*	1.99 ± 1.86	4.95 ± 4.95	36.1	0.240	0.315
*Viola patrinii*	3.64 ± 2.25	2.67 ± 1.62	49.5	0.008	0.971
*Poa pratensis*	3.62 ± 2.61	2.46 ± 1.07	47.1	0.051	0.853
*Anemone rivularis*	0.97 ± 0.62	3.66 ± 1.94	49.1	0.017	0.971
*Ranunculus japonicus*	6.99 ± 3.90	0.09 ± 0.05	34	0.271	0.247
*Trollius ranunculoides*	6.48 ± 6.08	2.67 ± 2.41	50.1	0.001	0.998
*Potentilla anserina*	19.75 ± 5.46	22.43 ± 7.73	48.5	0.034	0.912

**Table 5:** Proportion of consumption of plants with different root types by male and female plateau zokor.

Root Type	Male Plateau Zokor (%)	Female Plateau Zokor (%)	*U*	*r*	*p*
Rhizomatous root system type	19.67 ± 6.24	10.76 ± 2.60	34	0.270	0.247
Caespitose root system type	0.93 ± 0.50	0.28 ± 0.10	39	0.186	0.436
Taproot root system type	28.58 ± 9.68	50.59 ± 9.67	32	0.304	0.190
Fibrous root system type	12.32 ± 6.18	10.14 ± 5.01	50	0.002	0.997
Sucker root system type	0.33 ± 0.24	0.54 ± 0.45	41	0.152	0.529
Tuberous root system type	34.73 ± 9.16	27.30 ± 8.95	42	0.135	0.579
Others	3.44 ± 1.79	0.38 ± 0.13	29	0.355	0.123

**Table 6:** Trophic niche breadth and overlap of male and female plateau zokor.

	Male Plateau Zokor	Female Plateau Zokor	*p*
Trophic niche breadth	3.39 ± 0.51	2.58 ± 0.29	0.393
Trophic niche overlap	0.91		-

## Data Availability

The datasets used and/or analyzed during the current study are available from the corresponding author on reasonable request.

## Appendix A

All plant taxa included in the plant DNA barcoding database established in this study are listed in the table below.

**Table A1:** The set of plants collected in this study.

Family	Genus	Species
Cyperaceae	*Kobresia*	*Kobresia humilis*
		*Kobresia capillifolia*
	*Carex*	*Carex aridula*
Poaceae	*Elymus*	*Elymus nutans*
	*Poa*	*Poa pratensis*
	*Koeleria*	*Koeleria macrantha*
Asteraceae	*Ligularia*	*Ligularia virgaurea*
	*Saussurea*	*Saussurea pulchra*
		*Saussurea sungpanensis*
		*Saussurea longifolia*
	*Leontopodium*	*Leontopodium souliei*
	*Taraxacum*	*Taraxacum mongolicum*
	*Artemisia*	*Artemisia frigida*
	*Anaphalis*	*Anaphalis lactea*
Ranunculaceae	*Anemone*	*Anemone rivularis*
		*Anemone imbricata*
		*Anemone trullifolia*
	*Ranunculus*	*Ranunculus japonicus*
	*Gymnaconitum*	*Gymnaconitum gymnandrum*
	*Trollius*	*Trollius ranunculoides*
	*Aconitum*	*Aconitum pulchellum*
Fabaceae	*Oxytropis*	*Oxytropis kansuensis*
	*Medicago*	*Medicago ruthenica*
	*Gueldenstaedtia*	*Gueldenstaedtia verna*
	*Astragalus*	*Astragalus polycladus*
Orobanchaceae	*Pedicularis*	*Pedicularis kansuensis f. albiflora*
		*Pedicularis kansuensis*
Gentianaceae	*Gentiana*	*Gentiana macrophylla*
		*Gentiana aristata*
	*Gentianopsis*	*Gentianopsis paludosa*
	*Swertia*	*Swertia bimaculata*
Rosaceae	*Potentilla*	*Potentilla anserina*
		*Potentilla discolor*
Euphorbiaceae	*Euphorbia*	*Euphorbia esula*
Lamiaceae	*Elsholtzia*	*Elsholtzia densa*
	*Ajuga*	*Ajuga lupulina*
	*Phlomoides*	*Phlomoides umbrosa*
		*Phlomoides rotata*
Plantaginaceae	*Plantago*	*Plantago depressa*
Apiaceae	*Sphallerocarpus*	*Sphallerocarpus gracilis*
	*Carum*	*Carum carvi*
	*Bupleurum*	*Bupleurum chinense*
Mazaceae	*Lancea*	*Lancea tibetica*
Geraniaceae	*Geranium*	*Geranium pylzowianum*
Primulaceae	*Lysimachia*	*Lysimachia maritima*
Violaceae	*Viola*	*Viola patrinii*
Boraginaceae	*Microula*	*Microula sikkimensis*
Caryophyllaceae	*Arenaria*	*Arenaria serpyllifolia*
Polygonaceae	*Bistorta*	*Bistorta vivipara*
